# Regulating Protein–RNA Interactions: Advances in Targeting the LIN28/Let-7 Pathway

**DOI:** 10.3390/ijms25073585

**Published:** 2024-03-22

**Authors:** Greater Kayode Oyejobi, Xiaodan Yan, Piotr Sliz, Longfei Wang

**Affiliations:** 1 Key Laboratory of Combinatorial Biosynthesis and Drug Discovery (Ministry of Education), School of Pharmaceutical Sciences, Wuhan University, Wuhan 430072, China; greateroyejobi@whu.edu.cn (G.K.O.); yanxd09@whu.edu.cn (X.Y.); 2 Department of Biological Chemistry and Molecular Pharmacology, Harvard Medical School, Boston, MA 02115, USA

**Keywords:** LIN28, let-7, miRNA, small-molecule inhibitor, oncogene, gene therapy

## Abstract

Originally discovered in *C. elegans*, LIN28 is an evolutionarily conserved zinc finger RNA-binding protein (RBP) that post-transcriptionally regulates genes involved in developmental timing, stem cell programming, and oncogenesis. LIN28 acts via two distinct mechanisms. It blocks the biogenesis of the lethal-7 (let-7) microRNA (miRNA) family, and also directly binds messenger RNA (mRNA) targets, such as IGF-2 mRNA, and alters downstream splicing and translation events. This review focuses on the molecular mechanism of LIN28 repression of let-7 and current strategies to overcome this blockade for the purpose of cancer therapy. We highlight the value of the LIN28/let-7 pathway as a drug target, as multiple oncogenic proteins that the pathway regulates are considered undruggable due to their inaccessible cellular location and lack of cavities for small molecule binding.

## 1. Introduction

**Figure 1:**
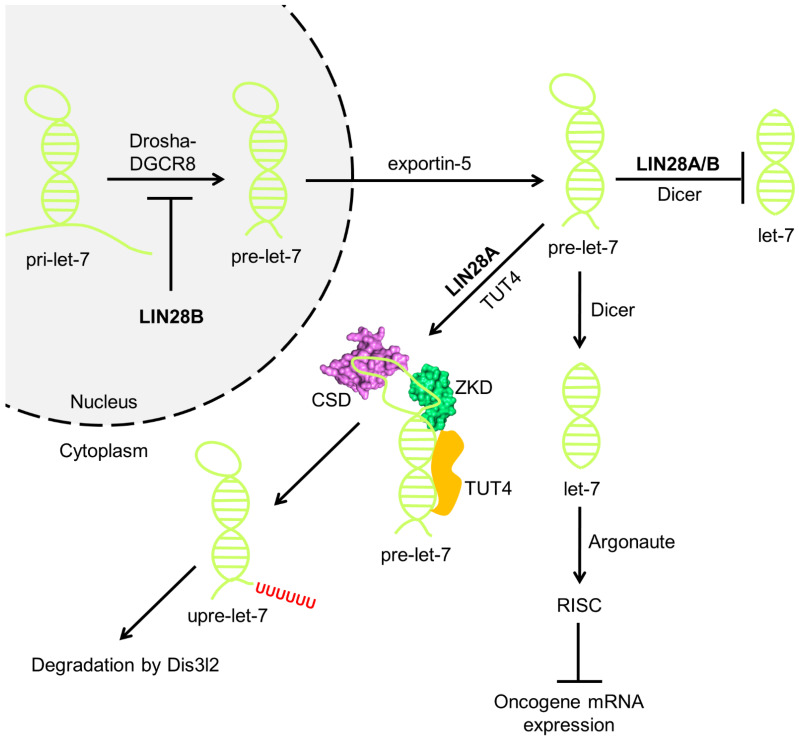
LIN28 Inhibits let-7 miRNA Biogenesis and Contributes to Cancer. In the nucleus, human LIN28B sequesters pri-let-7 and as a result, the Drosha-DGCR8 microprocessor fails to generate pre-let-7, thereby arresting let-7 biogenesis. LIN28A (CSD—cold-shock domain; ZKD—zinc knuckle domain) binds to pre-let-7 after its nuclear export. Given the cytoplasmic localization of LIN28B in some cell types, LIN28B inhibition of mature let-7 biogenesis can also occur in the cytoplasm. LIN28A/B recruits TUT4/Zcchc11, which oligouridylates pre-let-7 at its 3′ end. Oligouridylated pre-let-7 then undergoes exonuclease degradation by DIS3L2. LIN28A/B binding to pre-let-7 could interfere with Dicer cleavage, providing the mechanism for TUTase-independent repression. Without this inhibition, pre-let-7 undergoes processing by Dicer to yield mature let-7 miRNA, which constitutes part of RISC and inhibits multiple oncogenes through RNAi.

Mature microRNAs (miRNAs) are cytosolic short single-stranded RNAs (ssRNAs) of approximately 22 nucleotides (nts) in length. Generation of miRNAs requires processing by nuclear RNase Drosha followed by cytoplasmic RNase Dicer (Figure 1). In the nucleus, Drosha binds DGCR8 to form the microprocessor complex, which cleaves a long primary miRNA (pri-miRNA) into a characteristic hairpin-like structure known as pre-miRNA [1,2,3]. Exportin-5 then transports the pre-miRNA to the cytosol and Dicer trims the cytosolic pre-miRNA into a final ~22-nt miRNA [4,5,6,7]. The mature miRNA binds an Argonaute protein to form an RNA-induced silencing complex (RISC), which utilizes the miRNA as a guide to target complementary 3′-untranslated regions (3′-UTRs) of messenger RNAs (mRNAs) and suppress the expression of the mRNAs through diverse mechanisms, such as mRNA degradation and translational repression, a phenomenon termed RNA interference (RNAi) [8,9,10]. 

The lethal-7 (let-7) family of miRNAs, including let-7a-1, let-7a-2, let-7a-3, let-7b, let-7c, let-7d, let-7e, let-7f-1, let-7f-2, let-7g, let-7i, and miR-98 in humans, regulate development and act as tumor suppressors by targeting oncogenes including ras, HMGA, JAK, STAT3, NIRF, and c-Myc, which in their 3′ UTRs contain complementary sequences to the seed sequence GAGGUA in let-7 family members [11,12,13,14]. Because post-transcriptional repression of let-7 biogenesis by LIN28 generally upregulates tumorigenesis, LIN28 is regarded as an oncoprotein [15,16].

In vertebrates there are two LIN28 paralogs, LIN28A and LIN28B, which are aberrantly expressed in numerous human cancers, including T-cell lymphoma, neuroblastoma, breast cancer, and hepatoblastoma [15,17,18,19]. LIN28A and LIN28B utilize distinct mechanisms to block let-7 miRNA biogenesis (Figure 1) [20,21]. Whereas LIN28B sequesters let-7 pri-miRNA (pri-let-7) to disable its cleavage by the Drosha-DGCR8 microprocessor complex in the nucleus, LIN28A resides in the cytoplasm and acts on let-7 pre-miRNA (pre-let-7) through a terminal uridylyltransferase (TUTase)-dependent mechanism. More specifically, LIN28A recruits TUT4/Zcchc11 to uridylate pre-let-7 at its 3′ end [22,23,24]. Oligouridylated pre-let-7 (upre-let-7) cannot be processed by Dicer due to its elongated tail and will undergo degradation by the DIS3L2 exonuclease [25,26]. Lifting the blockade of let-7 miRNA biogenesis by LIN28 may therefore attenuate or prevent tumorigenesis.

It is important to mention that there exist contrasting viewpoints regarding the subcellular localization of LIN28B. For example, Piskounova et al. [20] showed that LIN28B possesses unique signals for localization in both the nucleolus and the nucleus, whereas Guo et al. [27], Molenaar et al. [28], and Hafner et al. [29] found that LIN28B is mainly located in the cytoplasm, indicating a potential for translocation into the nucleus in a manner dependent on the cell cycle. Thus, given that LIN28B localization varies in different cells, including cell lines where LIN28B is strictly cytoplasmic, its inhibition of let-7 biogenesis could still occur in the cytoplasm (Figure 1). Additional research is required to comprehensively elucidate the precise subcellular localization of LIN28B, which can potentially differ based on the specific cell type.

## 2. Structural Basis for Interaction of Let-7 MicroRNAs with LIN28

**Figure 2:**
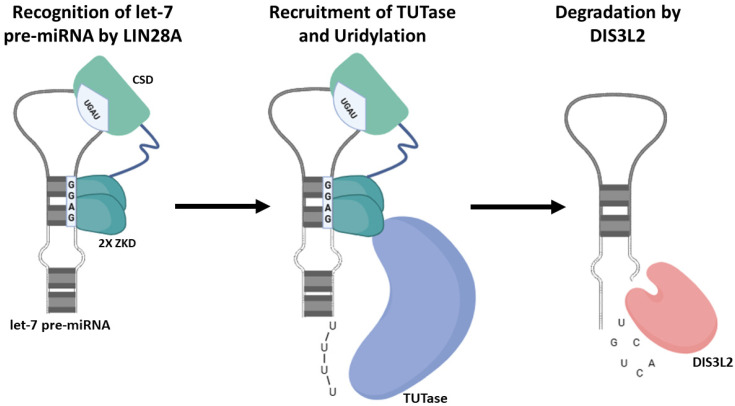
Involvement of LIN28A CSD and ZKD in let-7 pre-miRNA binding and processing. Using their CSD and ZKD binding elements, let-7 miRNA precursor recruits LIN28A, resulting to their 3ʹuridylation by TUTase and eventual degradation by DIS3L2.

To identify the specificity of the interaction of let-7 microRNA with LIN28, some structural data have been obtained. One major mechanism by which LIN28 selectively modulates let-7 microRNAs is based on the involvement of LIN28 binding domains. LIN28 contains two RNA binding domains, namely an N-terminal cold-shock domain (CSD) and zinc knuckle domain (ZKD) that contains two zinc knuckles (CCHC-type) [30]. Both the CSD and ZKD recognize two distinct regions of the RNA and have been shown to have roles to play in RNA binding and let-7 processing (Figure 2) [31,32,33,34].

By identifying the structures of three LIN28A–pre-let-7 complexes, Nam et al. [35] reported a bipartite interaction between LIN28A and its let-7 family partners. They discovered that in the central stem-loop structure in pre-let-7, the CSD inserts into the loop at one end, and the two ZKD modules recognize a GGAG motif at the other end. Without compromising affinity or specificity, the flexible linker connecting the CSD and the two ZKD molecules can accept different sequences and lengths among the LIN28-regulated let-7 family members. When linked together, these domains are adequate to limit let-7 processing. There are two possible paths for processing pre-let-7 after LIN28 is bound. First, as LIN28 bends GGAG and positions itself in a certain conformation on one of the strands, it may function as a “wedge” to melt a portion of the double-stranded mature area. Therefore, Dicer may be unable to appropriately identify its substrate. Second, because of the position of the zinc knuckle binding domains, the volume of the ZKD, and the location of its N terminus, which the interdomain linker would have to cross in order to reach the CSD, LIN28 is likely to collide with the Dicer dsRNA-binding domains and obscure one of the cleavage sites.

Furthermore, Loughlin et al. [36] sought to understand how LIN28B specifically recognizes pre-let-7 terminal loops by determining the structure of the ZnF domains of LIN28 bound to RNA. The obtained solution’s structure reveals a somewhat degenerate 5′-NGNNG-3′ consensus-binding sequence that permitted the ZnFs of LIN28 to recognize all pre-let-7 miRNAs. The structure, which showed the interacting elements in the RNA and the protein, enabled the definition of a new consensus motif (5′-NGNNG-3′). Thus, by specifically recognizing these motifs, the ZnFs of LIN28 are sufficient for selective recognition of the pre-let-7 family [37].

While the zinc knuckle domain (ZKD) binds a GGAG-like element in the precursor to coordinate LIN28A/B recognition, the cold-shock domain (CSD) was shown by Ustianenko et al. [38] to recognize a (U)GAU motif. More importantly, they found that this motif partitions the let-7 microRNAs into two subclasses, precursors with both CSD and ZKD binding sites (CSD+) and precursors with ZKD but no CSD binding sites (CSD). On further study, they demonstrated that CSD binding sites amplify the regulatory effects of LIN28. Specifically, LIN28 in vivo recognition, and subsequent 3ʹ uridylation and degradation, of CSD+ precursors is more efficient, leading to their stronger suppression in LIN28-activated cells and cancers.

Summarily, LIN28 CSD and ZKD recognize distinct sequence motifs, and are both required for high-affinity interactions of LIN28 with let-7 pre-miRNAs.

## 3. Mechanistic Studies on the Regulation of Cancer Progression via the LIN28/Let-7 Axis

**Figure 3:**
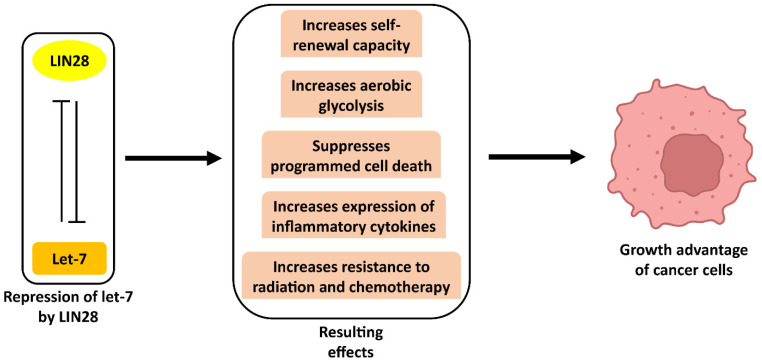
Some effects of the repression of let-7 by LIN28 on cancer cells.

LIN28A, an RNA-binding protein and LIN28B, its homolog, play major roles in cell growth and germ lineage [39]. LIN28A and LIN28B control gene regulatory networks through multiple mechanisms, the best-studied being the regulation of LIN28 on let-7 [21,40]. Regulation via the LIN28/let-7 axis is such that LIN28 and let-7 have opposite effects on developmental progression [41]. In order to promote differentiation programming, each of the let-7 family members decrease expression of genes that promote stemness, proliferation, and migration, whereas LIN28A/B derepresses these genes in a let-7-dependent way to maintain a pluripotent phenotype [42]. This provides a let-7-dependent mechanism of oncogene upregulation; as such, the LIN28/let-7 axis has been shown to regulate cancer development in various ways (Figure 3). In this section, we review different let-7-dependent mechanisms of LIN28A/B function across different tumor types. 

### 3.1. Modulation of Self-Renewal Capacity of Cancer Stem Cells

Cancers may arise from rare self-renewing tumor-initiating cells (T-IC), and miRNAs can regulate cell-fate decisions [43,44]. Surprisingly, studies have shown that let-7 can control the tumorigenicity and self-renewal of breast cancer cells. Yu et al. [45] compared miRNA expression in self-renewing and differentiated cells from breast cancer lines, and in breast BT-IC and non-BT-IC from 1° breast cancers. They found that let-7 miRNAs were markedly reduced in BT-IC and increased with differentiation. Low let-7 helped maintain the undifferentiated status and proliferative potential of mammospheric cells from a cell line and of 1° tumor BT-IC. These results suggest a mechanism whereby repression of let-7 by LIN28A and LIN28B may help to confer self-renewal capacity on cancer stem cells (CSC). Similarly, Yang et al. [46] showed that the self-renewal and differentiation of mammary gland epithelial progenitor cells are regulated by a LIN28/let-7 loop. They discovered that a LIN28B/let-7 regulatory loop regulates ALDH1+ cancer stem cells and that LIN28B maintains the population of ALDH1+ tumor cells by controlling let-7. In another study, Albino et al. [47] demonstrated that a crucial factor in the cell transformation and proliferation of prostate CSC was the deregulation of the LIN28A/B–let-7 axis with decreased let-7 microRNA synthesis. They found links between the LIN28/let-7 microRNA axis, the CSC subpopulation in prostate cancer, and the ETS transcription factor ESE3/EHF. The findings showed that a tumorigenic and stem-like phenotype in prostate cancer is promoted by the activation of the LIN28/let-7 axis caused by the loss of ESE3/EHF.

### 3.2. Regulation of Aerobic Glycolysis to Promote Cancer Progression

Altered metabolism plays an important role in promoting malignant tumor characteristics. Given that cancer-specific metabolism is mainly responsible for the growth advantage of cancer cells [48,49], studies have been carried out to try to understand the mechanisms by which the LIN28/let-7 axis regulates glucose metabolism. In a study by Ma et al. [50], it was found that LIN28A and LIN28B enhance aerobic glycolysis while let-7 suppresses it by targeting pyruvate dehydrogenase kinase 1 (PDK1). This finding demonstrates a novel pathway to mediate aerobic glycolysis of cancer cells even in ambient oxygen levels, independent of hypoxia or hypoxia-inducible factor-1 (HIF-1). This discovery indicates that the LIN28/let-7 axis promotes the growth of cancer by facilitating aerobic glycolysis. Another study by Gibadulinova et al. [51] demonstrated that an increase of let-7 miRNAs in CAIX-suppressed cells simultaneously caused a decrease in LIN28A/B protein levels, along with downstream metabolic pathways (PDK1), eventually resulting in the attenuation of glycolysis. Overall, they demonstrated that during CAIX-mediated adaptation to hypoxia and acidosis in carcinogenesis, glycolytic metabolism is increased and stem cell markers are expressed more when the LIN28/let-7 axis is regulated by CAIX. Ackermann et al. [52] also reported a cancer-type metabolic shift induced via a LIN28B/let-7 axis in mice. Specifically, liver-enriched inhibitory protein (LIP) activates LIN28B through repression of the let-7 microRNA family. Moreover, they showed that transgenic mice overexpressing LIP have reduced levels of let-7 and increased LIN28B expression, which is linked to skin hyperplasia and metabolic reprogramming, as evidenced by primary bone marrow cells. This work demonstrates that LIP is both a regulator of the let-7/LIN28B regulatory circuit and an inducer of cancer type metabolic reprogramming.

### 3.3. Mediation of Cancer Cell Death and Evasion of Immune Destruction

The potential roles and underlying mechanisms of the LIN28/let-7 axis in apoptosis during cancer has been demonstrated. Using BGC-823 gastric cancer cells, Song et al. [53] reported that the overexpression of LIN28A was inversely correlated with the downregulated expression of let-7a, and markedly suppressed the proliferation, migration, and cell cycle progression, and induced apoptosis. Consistently, Zhang et al. [54] showed that silencing LIN28A/B promotes apoptosis in colorectal cancer cells by upregulating let-7c targeting of antiapoptotic BCL2L1. Furthermore, studies have shown that cancer cell immune evasion may be regulated via the LIN28/let-7 Axis. A study by Chen et al. [55] demonstrated that let-7 post-transcriptionally suppressed programmed death ligand-1 (PD-L1), which is a transmembrane immune protein that interacts with the T-cell inhibitory receptor programmed cell death protein-1 (PD-1). The upregulation of LIN28A/B in most cancer cells results in the inhibition of the biogenesis of let-7, thus promoting PD-L1 expression. This regulation of PD-L1 suggests that the LIN28/let-7 loop can affect cancer progression.

### 3.4. Mediation of Tumor-Associated Inflammation

Studies have suggested a strong association between inflammation and different types of cancer, and inflammatory molecules can provide growth signals that promote the proliferation of malignant cells [56]. Particularly, the transcription factor NF-κB that regulates the expression of antiapoptotic genes and activates different proinflammatory cytokines and chemokines, seems to be a key molecular link between inflammation and oncogenesis initiation and progression [57,58]. Iliopoulos et al. [59] reported that NF-κB mediated an inflammatory response that directly activates LIN28B transcription and rapidly reduces let-7 microRNA levels. They showed that let-7 directly inhibits IL6 expression, resulting in higher levels of IL6 than achieved by NF-κB activation. This suggests that the LIN28A/B and let-7 loop is a key switch linking inflammation to cell transformation.

### 3.5. Regulation of Radio- and Chemo-Resistance in Cancer

Resistance to radiation and chemotherapy is a major obstacle for the effective treatment of cancer, and the LIN28/let-7 axis has been shown to regulate this resistance. Lv et al. [60] demonstrated the association of LIN28A/B with resistance to paclitaxel, a first-line chemotherapy drug. In their study, in comparison to the MCF7, Bcap-37, or SK-BR-3 cancer cell lines, which exhibited low levels of LIN28 expression, the T47D cancer cell line, which expresses LIN28 abundantly, was more resistant to paclitaxel. The sensitivity to paclitaxel treatment was improved in LIN28A high-expression T47D cells when LIN28A was knocked down; however, stable expression of LIN28 in breast cancer cells substantially reduced the sensitivity to paclitaxel treatment, leading to a considerable increase in paclitaxel IC50 values. The same research group also reported that the T47D cancer cell line is more resistant to radiation than MCF7, Bcap-37 or SK-BR-3 cancer cell lines [61]. In another study by Yin et al. [62] on radio- or chemo-resistance in NSCLC cells, it was demonstrated that by controlling the NSCLC cells’ capacity for single-cell proliferation, let-7 downregulation and LIN28A/B upregulation enhanced resistance to radiation or cisplatin. The LIN28A/B–let-7 axis was also found to modulate the radiosensitivity of cancer cells through the activation of K-Ras. In a study by Oh et al. [63], the inhibition of LIN28A/B resulted in the overexpression of let-7a, which subsequently attenuated K-Ras expression and radiosensitized A549 and ASPC1 cells.

## 4. Pharmacological Modulation of the LIN28/Let-7 Axis for Cancer Therapy

**Figure 4:**
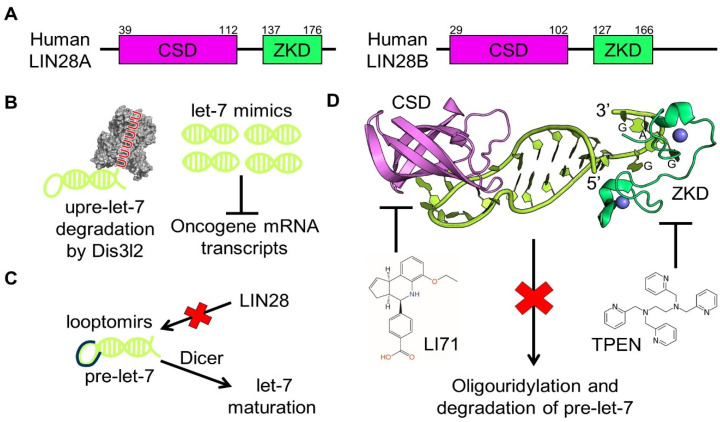
Pharmacological inhibition of LIN28-driven oncogenesis. (**A**) Domain architecture of human LIN28A and LIN28B, which share an N-terminal CSD (magenta) and a C-terminal ZKD (green) The residue numbers are denoted atop. (**B**) Let-7 mimics enhance tumor suppression by replenishing let-7, the level of which is decreased by LIN28-mediated oligouridylation and degradation by nuclease DIS3L2 (grey) (PDB ID: 4PMW). (**C**) Looptomirs hinder (red cross) LIN28 binding to pre-let-7 without blocking downstream pre-let-7 processing by Dicer, thereby promoting let-7 maturation. (**D**) Small molecules LI71 and TPEN inhibit (red cross) LIN28 by targeting its CSD and ZKD, respectively. Crystal structure of human LIN28A in complex with pre-let-7f-1 (yellow) (PDB ID: 5UDZ). Zn^2+^ ions (blue) are visible near the ZKD. The GGAG motif in pre-let-7f-1 is indicated near its 3′ end.

To suppress let-7 biogenesis, LIN28 sequesters pri-let-7 or induces pre-let-7 degradation. In return, let-7 can target the 3′ UTR of LIN28 mRNA to downregulate LIN28 expression, creating a double-negative feedback loop [64,65]. Given that let-7 is tumor-suppressive, increasing let-7 levels will likely attenuate tumorigenesis, especially that driven by LIN28. Indeed, administration of let-7 prevents tumor formation in a mouse model of non-small-cell lung cancer (NSCLC) and LIN28B knockout and knockdown effectively upregulate let-7 levels and curb Wilms tumors and hepatoblastomas in mice [66,67,68,69,70]. These studies have established the genetic and epigenetic basis for modulating the LIN28B/let-7 axis for cancer therapy. Current strategies to upregulate let-7 activity trifurcate into gene therapy with let-7 mimics, short modified oligoribonucleotides known as looptomirs, and small-molecule inhibitors of LIN28 (Figure 4). Here, we briefly discuss advances in let-7 mimics and looptomirs and focus on the discovery and mechanisms of several LIN28 inhibitors.

### 4.1. Gene Therapy

Because let-7 is tumor-suppressive and has multiple oncogene targets, using gene therapy to replenish let-7 has potential as a cancer therapy [71,72,73] (Figure 4B). As a proof of concept, Wang et al. showed that let-7i mimics of 22 base pairs (bps) in length potently inhibit the growth and migration of A549 cells, results that are consistent with those from Ling et al., who demonstrated that let-7a-mimic treatment hinders epithelial–mesenchymal transition, cell mobility, and expression of VEGF-C and MMP9 in esophageal squamous cell carcinoma (ESCC) cells [74,75]. Taken together, these studies raise hope for the development of anti-cancer therapeutics using let-7 mimics and shed light on a general direction of engineering miRNA analogs for tuning biological pathways. However, there remains the issue of how to deliver small RNA molecules specifically and effectively, including miRNA mimics. More studies are therefore needed to develop successful delivery methods.

### 4.2. Looptomirs

Antisense oligoribonucleotides are another class of let-7-targeting therapeutics (Figure 4C). Using an RNA-based, enzyme-linked immunosorbent assay (ELISA), Roos et al. described a 13-nt looptomir, dubbed L29-13, that binds to pre-let-7a with high affinity and without blocking downstream Dicer [76]. L29-13 competitively antagonizes LIN28A/B recruitment and inhibits cancerous proliferation in hepatocellular carcinoma (HCC)-derived Huh7 and HepG2 cells, laying the groundwork for the development of small molecules that target the LIN28/pre-let-7 interaction. Looptomirs and small-molecule inhibitors both aim to disrupt LIN28/pre-let-7 interaction and promote let-7 generation, the former preferably targeting pre-let-7, the latter LIN28. Although more let-7-specific looptomirs await development, several studies have demonstrated the ability of looptomirs to modulate maturational processing of miRNAs, highlighting looptomirs as a promising strategy in LIN28/let-7-targeting cancer therapy [77,78].

### 4.3. Small-Molecule Inhibitors

Following the discovery of let-7 mimics and looptomirs as potential therapeutic agents, researchers have reported several small-molecule inhibitors of LIN28, including Compound 1632 and Compound 1, discovered through fluorescence resonance energy transfer (FRET)-based screens [79,80,81,82,83]. The molecular mechanisms of inhibition for these compounds are not yet well understood, making it difficult to optimize these compounds to improve potency and reduce toxicity. 

Biochemical and structural efforts allowed us to make significant progress towards the discovery and mechanistic elucidation of LIN28 inhibitors (Table 1). Crystal structures of LIN28/pre-let-7 complexes demonstrate that the N-terminal cold-shock domain (CSD) and the C-terminal Cys-Cys-His-Cys (CCHC) zinc knuckle domain (ZKD) support a bipartite RNA-protein interaction, and therefore establish the molecular basis for pre-let-7 suppression (Figure 4A,D) [37,84]. The CSD binds to and structurally remodels pre-let-7 at its terminal loop, whereas the ZKD specifically binds to a conserved GGAG motif abutting the terminal loop (Figure 4D) [35,36,37,85]. Both domains of LIN28 and the GGAG motif of pre-let-7 are required to block LIN28-mediated recruitment of Zcchc11/TUT4 and pre-let-7 processing by Dicer. Although the high-affinity CSD/pre-let-7 interface is larger than the corresponding ZKD/pre-let-7 interface, mutations in either the CSD (such as F73A, G90A, and G119A) or ZKD (such as Y140A) restore pre-let-7 maturation into let-7, demonstrating that targeting the domains with small molecules, individually or in concert, has therapeutic potential [35,37].

Using a fluorescence polarization assay, Wang et al. identified six small molecules that inhibit LIN28A/B–pre-let-7 binding and LIN28A/B-mediated oligouridylation of pre-let-7 [86]. Among the six inhibitors, LI71 and TPEN were extensively characterized and exhibited different molecular mechanisms. While LI71 acts by competing for the RNA-interacting site in the CSD, TPEN destabilizes the ZKD by chelating ZKD-bound Zn^2+^, changing the conformation of ZKD. Direct interaction between LI71 and the LIN28 CSD was detected by saturation transfer difference (STD) spectroscopy, and mutational analysis revealed that residue K102 of the CSD contributes to binding of LI71 to CSD. The fact that LI71 downshifts the melting temperature of the LIN28/pre-let-7 complex by 3 °C provides further evidence for competitive binding. A crystal structure of LIN28 with bound LI71 will further elucidate the LI71 mechanism of action. TPEN, by contrast, is a Zn^2+^ chelator in cells and thus may capture LIN28 ZKD-bound Zn^2+^ and induce destabilizing conformational changes. Indeed, residues in the zinc knuckles, such as C139 and C161, and in the GGAG-binding surface, such as Y140, are drastically rearranged following TPEN treatment, shown using ^1^H-^15^N heteronuclear single quantum coherence (HSQC) spectroscopy. With a low in vitro IC_50_ of 2.5 μM, TPEN is highly toxic to cells and, therefore, its cellular efficacy awaits evaluation. In comparison, LI71 has an IC_50_ of 50–100 μM in human leukemia and mouse embryonic stem cells. 

Compound 1, identified by Lim et al., and LI71 are similar because both compounds contain a benzoic acid moiety, suggesting that this group may represent a molecular scaffold for LIN28 inhibitors [83,86]. Consistently, 5-(methylamino)nicotinic acid (MNA), which consists of a benzoic acid-like moiety and minimal other groups, also blocks LIN28A/B activity in vitro and in HeLa cells, albeit with relatively high IC_50_s [86]. This shared feature raises hopes for fragment-based drug development using MNA as a minimal scaffold, and for the design of more potent benzoic acid analogs that inhibit LIN28. 

**Table 1:** Pharmacological strategies to modulate the LIN28/let-7 axis.

Strategy	Selected Agent	Mechanism of Action	Cellular Effect	Reference
Let-7 mimics	Let-7i mimic	Restoration of let-7i level	Inhibition of the growth and migration of lung cancer cells	[74]
	Let-7a mimic	Restoration of let-7a level	Suppression of Wnt signaling in esophageal squamous cell carcinoma (ESCC) cells	[75]
Looptomirs	L29-13	Antagonization of LIN28A docking at pre-let-7a-2	Restoration of let-7 synthesis and inhibition of growth and clonogenic potential in hepatocarcinomatous cells overexpressing LIN28B	[76]
Small-molecule inhibitors	Compound 1	Prevention of LIN28A binding to pre-let-7a-1 through unknown molecular mechanism	Enhancement of let-7 production in LIN28-expressing cancer cells (LIN28A and LIN28B)	[83]
	6-hydroxy-DL-DOPA	Prevention of LIN28A binding to pre-let-7g through unknown molecular mechanism	N/A	[87]
	SB/ZW/0065	Prevention of LIN28A binding to pre-let-7g through unknown molecular mechanism	N/A	[87]
	Compound 1632	Prevention of LIN28B binding to pre-let-7a-2 through unknown molecular mechanism	Reduction in let-7 level and tumor sphere formation in LIN28-expressing cancer cells; induction of mouse embryonic stem cell differentiation	[88]
	TPEN	Chelation of LIN28A ZKD-bound Zn^2+^ and destabilization of LIN28A ZKD	N/A	[86]
	LI71	Competition for the pre-let-7f-1 interaction site of LIN28A CSD	Suppression of LIN28 activity and restoration of mature let-7 level in leukemia cells (LIN28B) and embryonic stem cells (LIN28A)	[86]
	Gossypol (LI11)	Prevention of LIN28A binding to pre-let-7f-1 through unknown molecular mechanism	N/A	[86]
	MNA (LI101)	Putatively by competition for the pre-let-7f-1 interaction site of LIN28A CSD	Suppression of LIN28 function in HeLa cells expressing LIN28A or LIN28B	[86]
	Trisubstituted pyrrolinones (C902)	Putatively by competition for the pre-let-7f-1 interaction site of LIN28A CSD	Increase in mature let-7 levels in human choriocarcinoma cell line JAR cells	[81]
	Compound 4j (GG-43)	Prevention of LIN28 binding to pre-let-7 through unknown molecular mechanism	Compound 4j barely showed any inhibitory activity in human choriocarcinoma cell line JAR cells	[82]

## 5. Outlook for LIN28/Let-7-Targeting Therapy

Although rationally designed and theoretically feasible, LIN28/let-7-targeting therapeutics may face significant challenges in clinical applications. It is known that double-stranded oligonucleotides exhibit poor cellular permeability and therefore transmembrane penetration of let-7 mimics may need to rely on engineered vehicles [89]. Commonly used gene delivery strategies to restore tumor-suppressing miRNAs or mimics include liposomes, non-viral vectors, and viral vectors [90,91,92,93]. Viral vectors are often problematic because they are time-consuming, costly, and immunogenic, and are nonspecifically integrated into the host genome. Systemic delivery of a let-7 mimic with a lipid emulsion formulation leads to efficient take-up by lung tissue and a drastic decrease in tumor burden in a Kras-activated autochthonous mouse model of NSCLC [94]. There also exist ample polymeric nanoparticle-based strategies for improving systemic circulation, local retention, cell permeability, and immunocompatibility of small-molecule cancer therapeutics [95,96]. These bioengineering approaches for drug delivery may prove valuable as more LIN28 inhibitors are being discovered.

Besides conquering delivery issues, one needs to strike a balance between potency and the safety of small-molecule inhibitors of LIN28. As a potent Zn^2+^ chelator, TPEN can nonspecifically compete for Zn^2+^ in cells and therefore exhibits high cytotoxicity. The observation that cancer cells express LIN28 at a higher level than normal cells, such that TPEN may more readily kill cancer cells, potentially alleviates this adverse effect, but structure-based drug design may be necessary to modify TPEN and improve its specificity to LIN28 before clinical application [97,98,99,100,101]. For such LIN28 inhibitors as LI71 [86] and Compound 1 [83], which are minimally toxic on cell lines, functional group modifications through medicinal chemistry may boost potency [102,103].

## 6. Conclusions

Many oncoproteins, including RAS and MYC, are considered undruggable because cavities for binding of small molecules are absent or subcellular localization inaccessible [104,105,106,107]. While suboptimal therapeutic efficacy may have dampened the passion for discovering inhibitors of many oncoproteins, the importance of miRNAs in oncogene regulation presents potential high-value targets for cancer therapy [108,109,110]. Therapeutic intervention at the miRNA level bypasses the undruggable proteins and provides an indirect means to downregulate oncogene expression. The three strategies described offer great promise for modulating the LIN28/let-7 axis, whether by utilizing let-7 mimics to induce the silencing of oncogenes by offsetting the loss of let-7 due to exonuclease degradation; using let-7-targeting looptomirs to promote let-7 maturation by preventing the docking of LIN28 to pre-let-7; or utilizing small-molecule inhibitors to hinder oncogenesis by disrupting the binding of LIN28 to pre-let-7.

Although this review has focused on let-7 miRNAs, LIN28 can also impact the levels of non-let-7 miRNAs. For instance, Nowak et al. [111] elucidated the regulatory role of LIN28A in modulating miRNA-9, underscoring the importance of considering these targets in therapeutic strategies targeting the LIN28 pathway. Similarly, Tan et al. [112] sheds light on the broader regulatory network governed by LIN28 by showing that LIN28B expression level is a key variable that sets the magnitude of protein translation. As molecular mechanisms of the LIN28/let-7 network continue to be elucidated and drug targets shift from oncoproteins to their miRNA regulatory machinery, successful therapeutic intervention will rely on synergistic advances in LIN28 structure-based drug discovery, pharmaceutical chemistry, and small-molecule drug and gene delivery.
